# Investigating how Explicit Contextual Cues Affect Predictive Sensorimotor Control in Autistic Adults

**DOI:** 10.1007/s10803-022-05718-5

**Published:** 2022-09-05

**Authors:** Tom Arthur, Mark Brosnan, David Harris, Gavin Buckingham, Mark Wilson, Genevieve Williams, Sam Vine

**Affiliations:** 1 Department of Sport and Health Sciences, College of Life and Environmental Sciences, University of Exeter, St Luke’s Campus, Heavitree Road, Exeter, EX1 2LU UK; 2 Centre for Applied Autism Research, Department of Psychology, University of Bath, Bath, BA2 7AY UK

**Keywords:** Autism, Prediction, Virtual Reality, Active inference, Uncertainty, Volatility

## Abstract

Research suggests that sensorimotor difficulties in autism could be reduced by providing individuals with explicit contextual information. To test this, we examined autistic visuomotor control during a virtual racquetball task, in which participants hit normal and unexpectedly-bouncy balls using a handheld controller. The probability of facing each type of ball was varied unpredictably over time. However, during cued trials, participants received explicit information about the likelihood of facing each uncertain outcome. When compared to neurotypical controls, autistic individuals displayed poorer task performance, atypical gaze profiles, and more restricted swing kinematics. These visuomotor patterns were not significantly affected by contextual cues, indicating that autistic people exhibit underlying differences in how prior information and environmental uncertainty are dynamically modulated during movement tasks.

**Supplementary Information:**

The online version contains supplementary material available at 10.1007/s10803-022-05718-5.

## Introduction

Individuals with autism spectrum disorder (ASD; hereafter “autistic people”; Kenny et al., 2016) can face a range of sensorimotor difficulties, such as clumsiness, sensory disturbances, poor postural control, and issues with eye-hand coordination (see Coll et al., 2020; Fournier et al., 2010; Gowen & Hamilton, 2013 for reviews). These difficulties may be associated with restricted personal independence (Jasmin et al., 2009), deleterious health outcomes (McCoy et al., 2016), and atypical socio-behavioural development (MacDonald et al., 2013; Sutera et al., 2007). However, there is a lack of evidence-based interventions that are proven to effectively tackle autism-related sensorimotor issues and their underlying causes (Colombo-Dougovito & Block, 2019). The present research aims to initiate this line of enquiry, by exploring whether movement-based skills in autistic people are enhanced following the provision of explicit prior information about an individual’s current sensory environment.

A growing body of research suggests that sensorimotor interventions in autism should be focusing on aspects of *predictive control*. Autism-related movement atypicalities often reside in the planning or anticipatory stages of an action (Cannon et al., 2021; Cavallo et al., 2021; Fabbri-Destro et al., 2009; Hughes, 1996; Schmitz et al., 2003) and appear underpinned by context-sensitive differences in the use of prior expectations (Arthur et al., 2020, 2021; Palmer et al., 2015). Relatedly, autistic participants display atypical responses to unexpected sensory cues, while coordinating visuomotor actions as if their surroundings are highly unpredictable or volatile (Arthur et al., 2021; Lawson et al., 2017). This suggests that autistic people may have difficulties forming stable predictions about their environment (Lawson et al., 2014; Van de Cruys et al., 2014). Indeed, predictive processing theories propose that higher-level beliefs (about uncertainty and stability) play a crucial role in regulating the dynamic weighting of top-down predictions and bottom-up sensory information (Mathys et al., 2014; Yu & Dayan, 2003). Given the increasing evidence that these mechanisms are atypical in autism (Palmer et al., 2017), interventions should look to help autistic people build flexible, contextually-appropriate predictions that can guide their sensorimotor actions.

A promising method for enhancing predictive sensorimotor control is to provide prior contextual information about the uncertain (and often complex) world that surrounds a task (see Haker et al., 2016). The notion of making conditions ‘more understandable’ is commonly advocated in the field, and studies show that autistic people can be explicitly cued or primed to process context in social cognition and perceptual discrimination tasks (e.g., Balconi et al., 2012; Cannon et al., 2021; Gowen et al., 2020; López et al., 2004; Plaisted et al., 1999; Vermeulen, 2015). From a mechanistic viewpoint, such an approach could particularly help individuals that have difficulties extracting the ambiguous contextual relationships that underpin dynamic sensorimotor interactions (e.g., autistic people: Qian & Lipkin, 2011; Van de Cruys et al., 2014). Indeed, a person’s ability to detect implicit and probabilistic regularities from the world around them can shape their higher-level beliefs about environmental uncertainty and stability (see Yon & Frith, 2021). So, by providing dynamic and explicit information about a task’s underlying probabilistic state, one may be able to enhance the context-sensitive modelling of sensorimotor predictions in this setting.

Nevertheless, the degree to which explicit contextual cues can augment autistic sensorimotor abilities remains unclear. There are a lack of studies examining autistic movement control within naturalistic tasks, and it is uncertain whether such difficulties reflect impairments in *making* accurate predictions (as suggested above) or differences in *modulating* actions according to predictions about world and body states. Crucially, the weighting of sensory information and prediction errors is dependent on an array of neuromodulatory functions that may be atypical in autism (i.e., physiological systems that regulate synaptic gain signalling across hierarchical neural networks; Lawson et al., 2014; Quattrocki & Friston, 2014; Van de Cruys et al., 2014). These include: phasic noradrenergic activity (Lawson et al., 2021; Yu & Dayan, 2003), dopamine-serotonin interactions (Friston et al., 2012), and signalling in the anterior cingulate cortex and cerebellum (Behrens et al., 2007; den Ouden et al., 2010; Palacios et al., 2021). Differences in these systems could lead to pathological neural gain and disproportionate receptiveness to sensory inputs (see Lawson et al., 2014), irrespective of any explicit contextual aids. Indeed, it has been found that some prediction-related atypicalities in autism persist even after individuals have been told about likely upcoming events (Balsters et al., 2017; Greene et al., 2019; Thillay et al., 2016).

Likewise, sensorimotor differences may relate to inherent psycho-behavioural traits that are largely stable and unreceptive to contextual cues. For instance, intolerance of uncertainty has been widely documented in autistic populations (Boulter et al., 2014; Vasa et al., 2018). Individuals with greater intolerance of uncertainty often experience adverse emotional reactions to unpredictable stimuli (Dugas et al., 1997). Though mechanistically distinct from higher-level state estimates (see Bervoets et al., 2021), recent data suggest that associated increases in anxiety could impair key predictive processing functions (e.g. volatility-related learning rate modulation, Lawson et al., 2021). Furthermore, sensory issues in autism correlate with apprehension about environmental change (Pickard et al., 2020; Wigham et al., 2015). Therefore, the degree to which autistic people benefit from explicit contextual information could also depend on trait differences in behavioural inflexibility and/or intolerance of uncertainty.

### The Present Research

**Fig. 1:**
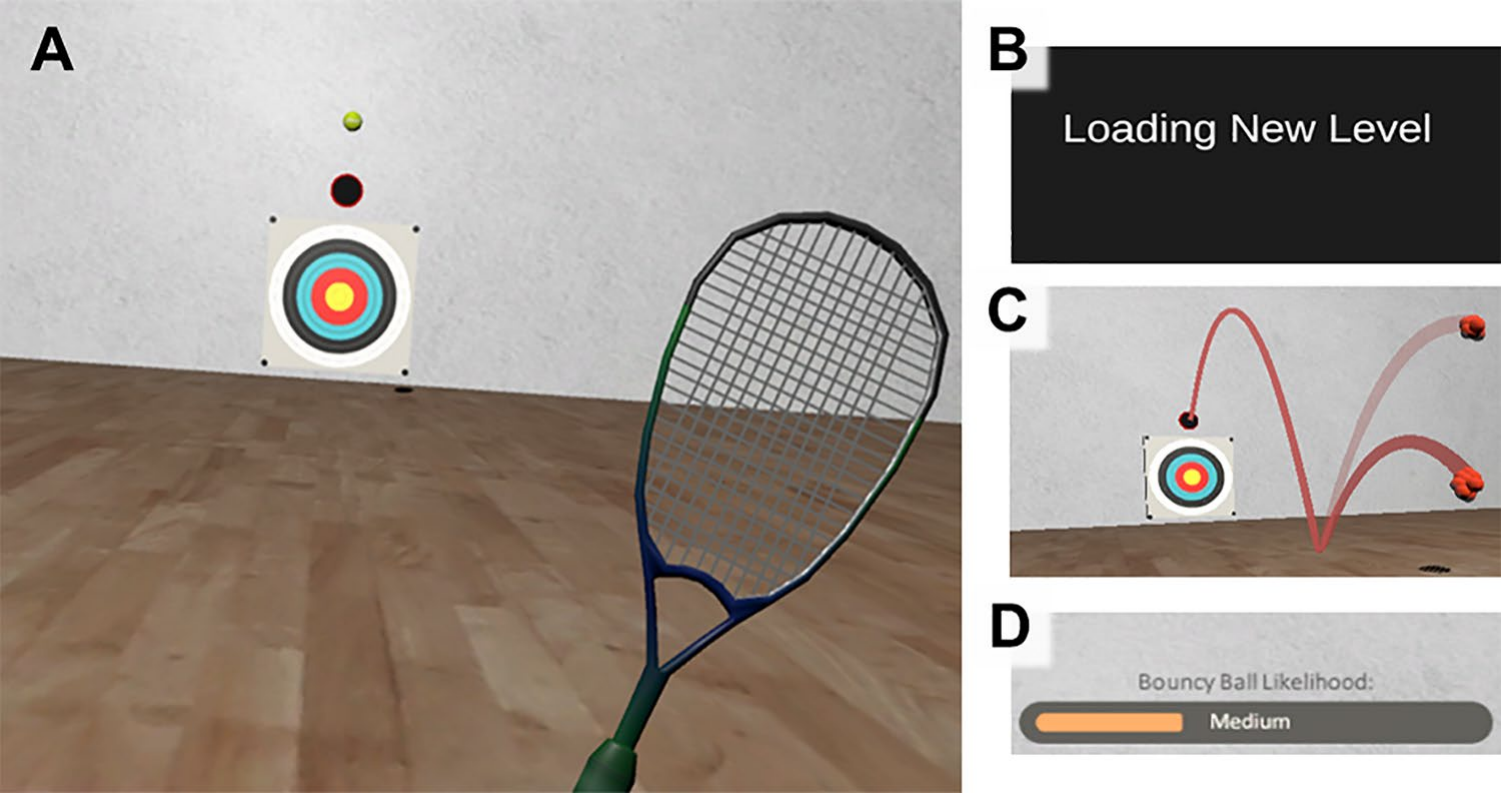
The Virtual Racquetball task. Participants were required to intercept balls that bounced with either normal or unexpectedly-high levels of elasticity. Gameplay footage of the virtual racquet, ball, and target is illustrated in panel (**A**). During control trials, participants received no explicit information about levels of ball bounciness and changing task conditions. However, three additional pieces of information were provided ahead of cued trials. Firstly, changes in task conditions were signalled using ‘game level’ transitions (**B**), which notified participants that they were about to enter a new environmental context. The proportion of normal and bouncy balls in each level were then projected in space using visual ‘hawkeye’ cues (**C**). A simulated ‘bounceometer’ on the front wall (shown in **D**) also confirmed whether a bouncy ball was ‘low’, ‘medium’, or ‘high’ in likelihood ahead of each trial. Together, these cues explicitly informed participants about dynamic task probabilities and environmental volatility. Supplementary Videos of the protocol can be found at: https://osf.io/5y48g/

This study examined the effects of explicit probabilistic cues on autistic sensorimotor control. Specifically, prior contextual cues were provided in virtual reality (VR) to help individuals make accurate, context-sensitive predictions during interceptive motor actions (Fig. 1). Research has shown that neurotypical individuals significantly benefit from this type of information during action-based tasks (Gray, 2015; Gredin et al., 2018; Navia et al., 2013). For instance, football goalkeepers display enhanced performance when provided with advanced information about an opponent’s most likely shooting direction (Navia et al., 2013). Although this type of intervention has not yet been tested in clinical populations, adaptive effects have been observed in autistic visual processing and motor imitation abilities when participants are explicitly cued towards goal-relevant contextual stimuli (Fulceri et al., 2018; Gowen et al., 2020; López et al., 2004; Soroor et al., 2021). Based on this evidence (and the studies discussed in the section above), we hypothesised that autistic people would show superior interceptive motor performances under cued versus non-cued trial conditions.

To further study the effects of explicit contextual cues on sensorimotor behaviours, we focused on participants’ hand movements and visual sampling responses. During interceptive tasks, a person’s movement patterns and gaze behaviours will be highly influenced by their prior expectations (see Arthur et al., 2021a; Mann, 2019). For instance, when an approaching ball is about to bounce, participants will direct their gaze away from its existing location towards its expected future position (Diaz et al., 2013; Mann et al., 2019), in what is referred to hereafter as the *predictive bounce fixation*. These behaviours are then updated in a context-sensitive manner. So, if ball elasticity (i.e., bounciness) is unexpectedly high and changeable, participants tend to increase the height and variability of their predictive bounce fixations, while reducing range of motion (ROM) in their swing (Arthur et al., 2021a). Although our previous work (Arthur et al., 2021b) illustrated that these predictive visuomotor adjustments are exhibited in both autistic and non-autistic populations, we observed that autistic people generally show higher, more variable fixations and more restricted swing movements than their neurotypical counterparts. These data imply that beliefs about environmental uncertainty and/or volatility are atypically high in these individuals. However, the explicit contextual cues in this study were designed to augment trial-by-trial state predictions, while reducing estimates of environmental uncertainty. Accordingly, we hypothesised that swing ROM would increase and the location of predictive bounce fixations would decrease in autistic participants from non-cued to cued conditions.

## Methods

We employed a virtual racquetball paradigm which has previously be used to investigate context-sensitive predictive action responses in neurotypical and autistic populations (Arthur et al., 2021a, 2021b; Diaz et al., 2013). In this task, participants use a VR handheld controller to intercept balls that either have normal or unexpectedly-high levels of bounciness. Environmental uncertainty and volatility are then systematically manipulated, through varying the likelihood of facing a normal or bouncy ball over time (in a manner that is consistent with computational studies in the field; e.g., Lawson et al., 2017). Our previous research has observed that autistic participants exhibit impaired sensorimotor performances in this task, especially under more volatile probabilistic conditions (Arthur et al., 2021b). However, the present study provided participants with prior information about the probability of facing a normal or bouncy ball over time (see Fig. 1). This would permit examination of whether sensorimotor control can be enhanced in autistic people through the use of explicit contextual cues.

### Participants

A total of 44 participants took part in the study (30 male, 14 female, 40 right-handed, mean age: 29 ± 7 years). 22 individuals had a formal diagnosis of ASD, as provided by an expert clinician according to DSM-IV (American Psychiatric Association, 2013) or ICD-10 (World Health Organisation, 2012) criteria, while the remaining sample were age- and gender-matched neurotypical controls. Though analysis was primarily interested in assessing cue-related changes in the ASD group, these neurotypical individuals could help elucidate any autism-related atypicalities shown between conditions. Inclusion criteria specified that participants were to be at least sixteen years of age. Participants were excluded if they had any co-occurring medical conditions or learning disabilities that are known to affect sensorimotor control. Eligible participants were naïve to the study aims and had no previous experience of playing VR-based racquet sports. All individuals in the ASD group scored above the clinical ‘screening cut-off’ of 26 on the 50-item Autistic Quotient (AQ; Baron-Cohen et al., 2001), with clinical characteristics proving highly consistent with previously reported values (Table 1; for normative data, see Baron-Cohen et al., 2001; Woodbury-Smith et al., 2005). Informed consent was obtained ahead of all study procedures in accordance with British Psychological Society guidelines. The study received approval from the Department of Sport and Health Sciences Ethics Committee (University of Exeter, UK) and the Department of Psychology Ethics Committee (University of Bath, UK).

**Table 1:** Participant characteristics. Group averages (and standard deviations) for all demographics and self-report measures that were recorded in the study

	ASD group	NT group
Sample size	*n* = 22	*n* = 22
Age	29.05 (7.52) years	29.55 (6.75) years
Gender	15 male, 7 female	15 male, 7 female
Dominant hand	21 right, 1 left	21 right, 1 left
AQ total score*	35.86 (5.37)	15.59 (7.96)
IUS-S score*	38.86 (9.98)	27.00 (10.14)

*ASD* autism spectrum disorder, *NT* neurotypical, *AQ* 50-item autistic quotient, *IUS-S* intolerance of uncertainty scale – shortened version,

*Denotes significant between-group difference (*p* < .001)

### Materials

The virtual racquetball environment was developed on the gaming engine Unity (Unity Technologies, San Francisco, CA) and is described at length in Arthur et al. (2021b). It was presented to participants on an HTC VivePro head-mounted display at 120 Hz (HTC Inc., Taoyuan City, Taiwan). This consumer-grade, high-precision VR system comprises two ‘lighthouse’ base stations, which record movements of the headset and hand controller at 90 Hz. The headset also contains an inbuilt eye-tracking system, which monitors users' gaze at 120 Hz with a spatial accuracy of 0.5–1.1. Participants were presented with a simulated 15 × 15 m racquetball court, which contained a circular target on its front wall (Fig. 1A). They were required to position themselves 9 m behind this location before attempting to hit virtual balls towards the middle of the target using a VR hand controller. This controller was displayed as a 0.6 × 0.3 × 0.01 m virtual racquet, and the balls resembled the appearance and size of those in ‘real-world’ tennis activities (Fig. 1A). All balls were launched from a height of 2 m, following three auditory tones, and would bounce 3.5 m in front of participants' prescribed starting position. Their trajectory passed through the midline of the room, which was 0.75 m to the right (for right-handers) or left (for left-handers) of this predetermined starting position.

For this study, the virtual environment was further adapted for the *cued* experimental condition. In these trials, participants would transition between five game ‘levels’, which provided explicit information about ball bounciness and environmental probabilities (Fig. 1). Level changes were signalled by an auditory tone and brief ‘loading screen’ (see Supplementary Video at: https://osf.io/5y48g/). Following this transition, participants would be transported into a new virtual room, to signal that their surrounding environment had changed. The front wall was visually identical for all levels, as were any ball bounciness- or goal-related action cues (e.g., the ball, floor, target and racquet). However, to emphasise that the underlying contextual probabilities had changed with each level transition, participants were presented with visual ‘hawkeye’ cues immediately after the loading screen (Fig. 1C). These illustrations projected the upcoming trajectory and ratio of normal and bouncy balls in each game level. Such ‘hawkeye’ cues were presented for 10 s and accurately represented the probabilistic ‘ground truth’ of a given level. They were accompanied by a visual indicator (referred to as the ‘bounceometer’), which explicitly stated whether the likelihood of getting a bouncy ball was low, medium, or high (Fig. 1D). Although this probabilistic information only reflected the statistical structure of a given *level* (i.e., they were not varied on trial-by-trial basis), they were presented for 3 s ahead of each *trial* in the cued condition. Such advanced information was not available in the practice or control conditions, nor in the final nine trials (i.e., game level) of the cued block.

Participants also completed the AQ (Baron-Cohen et al., 2001) and Intolerance of Uncertainty Scale–shortened version (IUS-S; Carleton et al., 2007). The AQ indexes five autistic-like traits: communication, imagination, social skills, attention switching, and attention to detail. Each trait subscale is scored out of ten and combined into an overall total (possible range: 0–50), which is said to index where an individual is situated on the ‘autism spectrum’ (Baron-Cohen et al., 2001). The resulting scores are continuous and normally-distributed in general populations, with clinical ASD viewed to reside at the extreme higher end of this continuum (Baron-Cohen et al., 2001; Ruzich et al., 2015). Conversely, the IUS-S is a 12-item questionnaire measuring intolerance of uncertainty, defined as the tendency of an individual to consider the possibility of a negative event occurring unacceptable, irrespective of the probability of occurrence (Carleton et al., 2007). Itemised statements are rated from 1 (not at all characteristic of me) to 5 (entirely characteristic of me) and combined into a total out of 60. Higher scores reflect greater intolerance of uncertainty, as is commonly reported in autistic populations (Boulter et al., 2014; Pickard et al., 2020; Wigham et al., 2015).

### Procedures

After providing written informed consent, participants were fitted with the head-mounted display and familiarised with the virtual environment. At this stage, the eye-tracker was calibrated over five gaze locations using the manufacturer’s built-in routine. Calibration was repeated before each experimental condition and upon any obvious displacement of the VR headset. Once familiarised with the virtual environment, participants then completed thirty baseline racquetball trials. Throughout this initial block, all virtual balls followed the same pre- and post-bounce trajectory, which were consistent with the effects of gravity (− 9.8 m/s^2^). Their speed remained fixed at − 9 m/s in the vertical plane (at the time of bounce), and elasticity was set at standard tennis ball levels (65%). Participants were instructed to hit balls towards the centre of the target, but that they would not be able to see or feel where they go after hitting them. This lack of feedback was used in all experimental trials to minimise confounding effects relating to motivation, communication skills, and task reward/error. Instead, a neutral ‘pop’ sound signalled when balls had made contact with the racquet, and any subsequent auditory and visual ball information was removed after this event.

**Fig. 2:**
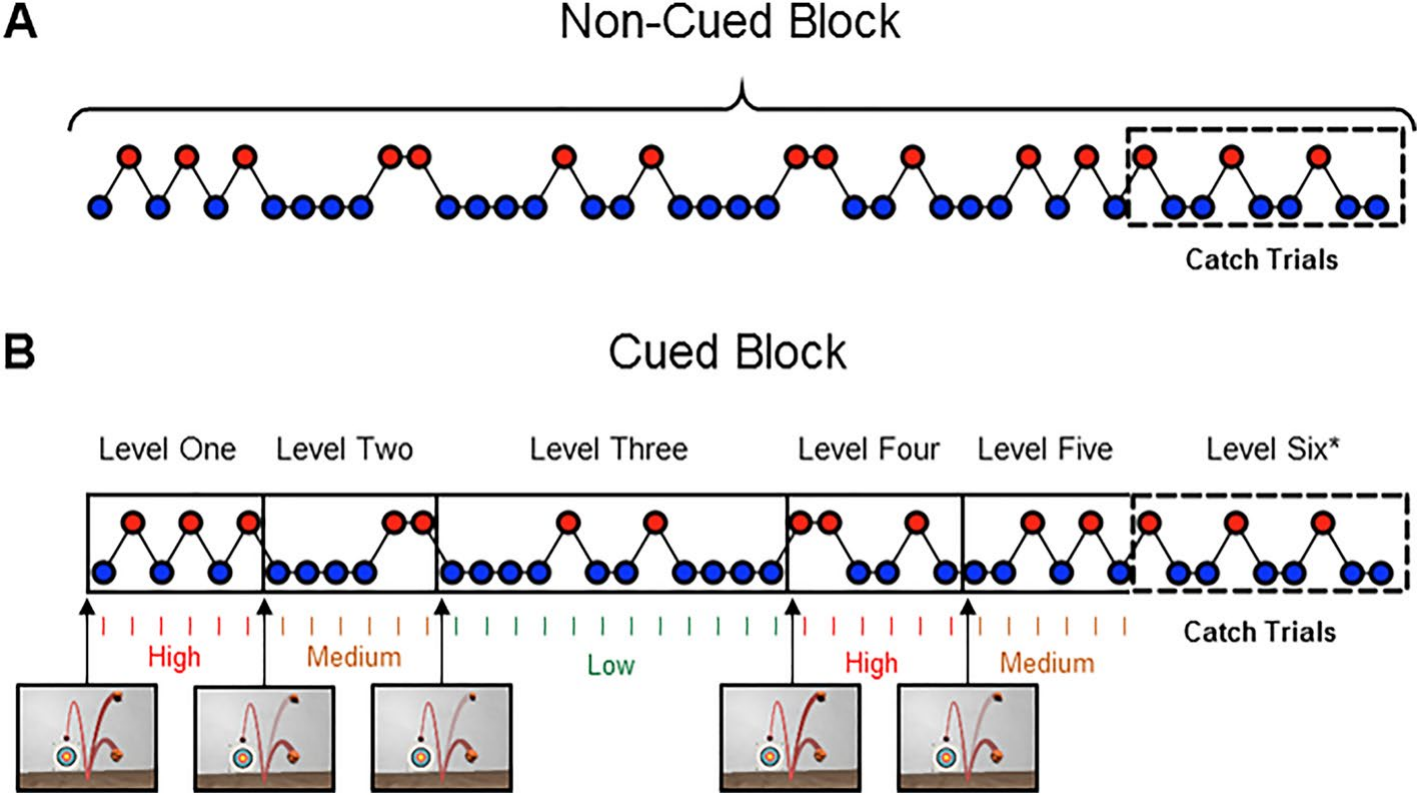
Schematic Illustration of the Experimental Protocol. Participants were presented with a series of balls that bounced with either normal (*blue circles*) or unexpectedly-high (*red circles*) levels of elasticity. Though trial order sequences were the same in each condition, Cued trials were separated into six game levels. Upon entering a new game level, participants received projected ‘hawkeye’ cues (see *arrows*). Each subsequent trial was then preceded by a visual indicator, which stated whether the likelihood of facing a bouncy ball was ‘low’ (17%), ‘medium’ (33%), or ‘high’ (50%) for this level. Conversely, balls in the non-cued condition were presented as one continuous sequence of trials, with no additional visual information. *Note that both blocks ended with nine catch trials that contained no explicit probabilistic cues

Following the initial baseline trials, participants performed two counterbalanced experimental conditions. In both of these blocks, ball bounciness was systematically varied over time to create unstable trial order sequences (illustrated in Fig. 2). While two-thirds of trials would contain ‘normal’ balls that were the same as those faced at baseline (and in real-world environments), a third contained ‘bouncy’ balls with unexpectedly high levels of elasticity (85%). This discernible change in post-bounce ball trajectory occurred without participant’s knowledge and would likely deviate away from any prior experiences obtained during ‘real-world’ actions. The *pre-bounce* ball speeds and trajectories were always the same as in baseline, meaning that the different type of balls were impossible to tell apart until they had made contact with the floor. Importantly, the probability of facing a normal ball changed every 6, 9 or 12 trials (between 83%, 67% and 50% likely). These unpredictably changeable post-bounce ball trajectories created a volatile environment, in which autistic people display impaired interceptive performances (Arthur et al., 2021b).

Participants were randomly allocated one of three possible trial order sequences (available at https://osf.io/5y48g/) which would be presented to them in both experimental conditions. For the *control* block, individuals did not receive any probabilistic information about likely ball bounciness and trials were presented as one continuous sequence (Fig. 2A). Instead, they were simply told that some balls may be more bouncy than others and that they should aim to hit as many of them as possible to the middle of the target.

Conversely, explicit information about situational probabilities were provided in the *cued* block. Here, visual cues indicated to participants both when ball bounciness probabilities were switching and how likely they were to face a ‘normal’ or ‘bouncy’ ball at any given time (see Fig. 2B). This direct provision of contextual priors has been proven to enhance visuomotor control in various neurotypical performance domains (e.g., Gray, 2015; Gredin et al., 2018; Navia et al., 2013). Participants were told that these visual cues would help show them where the balls are going to go and how likely they are to get a bouncy ball during each game level. They were not informed that the trial order sequences would be exactly the same in each experimental block.

The laboratory protocol generally lasted ~ 30 min in total. The two experimental conditions contained 45 trials each and were separated by a short break. The final 9 trials of each block contained identical visual information (i.e., no ‘hawkeye’ or ‘bounceometer’ cues) and thus provided a set of order-matched ‘catch’ trials for further examination (see Fig. 2).

### Data Analysis

Task performance was evaluated based on *interception rate*, which reflected the percentage of trials in which participants successfully hit the ball with their racquet. Kinematic variables were assessed using the positional data of the VR hand controller, which were extracted and then smoothed using a dual-pass, zero-phase Butterworth filter (frequency: 10 Hz; Franks et al., 1990). Specifically, analysis focused on the foreswing phase of interceptive actions, which started when the racquet first began to move forward and ended when it first made contact with the ball. In trials where participants failed to hit the ball, foreswing offset represents the final data point in which the ball’s depth position exceeded that of the racquet.

*Peak velocity* of the hand controller (in the direction of the target) was recorded from participants’ foreswing movements, as autistic participants displayed slower, more novice-like swing actions than neurotypical individuals in our previous work (Arthur et al., 2021b). *ROM* was assessed during this trial period to capture context-sensitive aspects of motor control. This outcome highlighted the maximum angular deviation between the VR headset and hand controller, as defined in the tranverse plane. Higher values would indicate that the hand had rotated to a greater degree around the body during the foreswing action. Conversely, decreases in ROM may signify that participants were ‘fixing’ movement degrees of freedom, a response which is typically prominent under volatile conditions and in autistic populations (Arthur et al., 2021b).

Eye tracking data were converted into ‘in-world’ angular vectors, as defined according to head-centred egocentric coordinates. Yaw and pitch values were smoothed using a three-frame median filter and then a second-order Butterworth filter (at 15 Hz; Cesqui et al., 2015). Since autistic participants employed anticipatory saccades in a similar manner to neurotypical individuals in our previous work (Arthur et al., 2021b), analysis only focused on predictive fixations. To extract this information, cleaned data were entered into a spatial dispersion algorithm (Krassanakis et al., 2014), which identified periods where gaze remained steady within a 3° area for a minimum of 100 ms. Trials where eye-tracking was temporarily lost (> 100 ms) or where there were > 20% of missing data were excluded. Subsequent analyses focused on the median onset time, mean duration, and average vertical position (mean pitch angle) of fixations that occur during (within 0.1 s), or immediately prior to, ball bounces in each trial. This *bounce fixation* is elevated when an individual predicts that ball elasticity likely to be higher (Diaz et al., 2013; Mann et al., 2019). Furthermore, trial-to-trial variability in this bounce fixation location is typically increased under volatile conditions (Arthur et al., 2021b).

*A-priori* power calculations indicated that a sample size of 40 would be sufficient to detect any moderate statistical effects in this study (*f* = 0.47), as estimated based on our previous data (Arthur et al., 2021b) and data in Lawson et al. (2017). Such calculations were conducted using G*Power 3.1 (Faul et al., 2007), with alpha set at *p* = 0.05 and power (*1 − β*) at 0.80. Poor motion tracking led to missing hand position data for one autistic participant. As such, they and their matched neurotypical counterpart were excluded from kinematic analyses (remaining *n* = 42). A separate pair of matched cases were removed from gaze analyses, due to frequent loss of eye-tracking signal (remaining *n* = 42). Data were deemed missing completely at random (Little’s MCAR test: *p* > 0.05). Two further autistic participants were identified as potential outliers in the interception rate data, but their average scores (45.56%, 54.44%) were consistent with previous values and the overall pattern of results was not affected by their inclusion. In these instances, conventions recommend that extreme values are *not* removed (Aguinis et al., 2013), as case exclusion may disregard important information relating to clinical difficulties. Consequently, no data were excluded for this variable.

All variables were entered into separate mixed-model ANOVAs, which studied main effects of *condition* (cued vs control) and *group* (ASD vs neurotypical), as well as *group-by-condition* interactions. Effect sizes were quantified using partial-eta squared. Significant effects were followed up using post-hoc *t-*tests, which were adjusted using the Bonferroni correction. Prior to running these mixed-model ANOVAs, manipulation checks examined whether predictive bounce fixations were sensitive to dynamic environmental probabilities. Here, dependent *t*-tests examined changes in bounce fixation pitch angles between baseline and control trials, to see whether unexpected and volatile manipulations of ball bounciness led to significant adjustments in predictive gaze positions. To explore relationships with autistic-like traits and intolerance of uncertainty, Pearson’s Correlation analysis examined associations between AQ scores, IUS-S scores, and all sensorimotor outcomes.

Interception rate data were positively skewed, with 10 participants intercepting 100% of balls. This outcome deviated from normality, along with peak swing velocity, fixation onset time, and fixation duration (all *p* < 0.05 for Shapiro–Wilk test). Mixed-model ANOVAs are robust to moderate deviations from statistical normality (Lix et al., 1996) and were still performed. However, Mann–Whitney *U* tests were employed for follow-up comparisons (using the Bonferroni correction) and Spearman’s Rho for assessing their correlations with AQ and IUS-S scores. Levene’s Test highlighted significantly different levels of variance for bounce fixation pitch measures (*p* < 0.05). No further assumptions were violated in relation to normality, sphericity, and homogeneity of variance. All statistical tests were conducted with alpha set at *p* < 0.05 and are reported alongside a Bayes Factor computation, which illustrates the strength of evidence in favour of the alternative/null hypotheses. Statistical procedures were undertaken using JASP 0.12.2, and the full study dataset is openly available at https://osf.io/5y48g/.

## Results

### Manipulation Checks

Manipulation checks showed a significant change in the height of predictive bounce fixations between baseline and control conditions (average pitch angle: *t*(39) = 6.73, *p* < 0.001, BF_10_ = 2.76 × 10^5^). As expected, volatile fluctuations in ball bounciness caused both groups to cast their gaze at a higher spatial location than at baseline, despite their being no explicit informational cues in either block of trials. These results confirm assumptions that participants would elevate their predictive bounce fixations when faced with unexpectedly bouncy balls and volatile trial conditions.

### Task Performance and Swing Kinematics

**Fig. 3:**
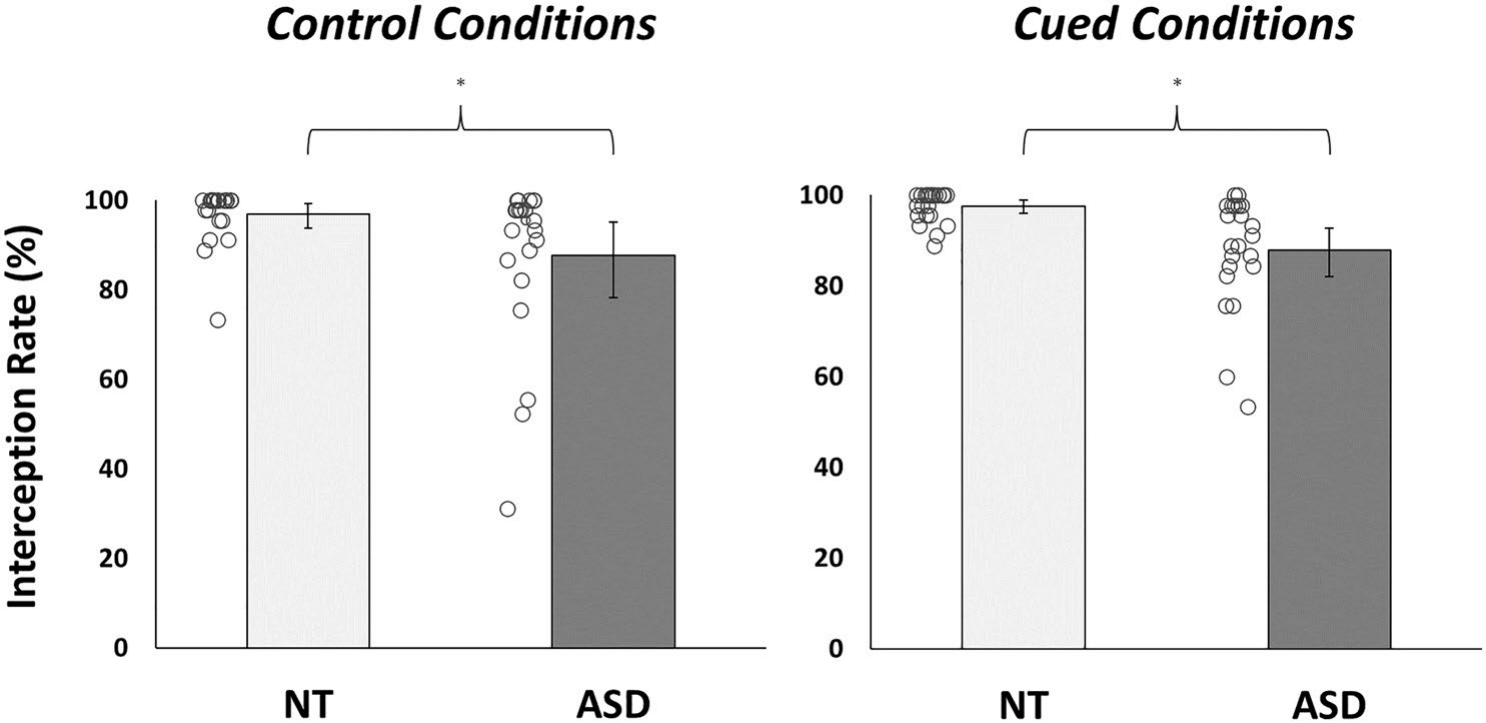
Task Performance. The proportion of balls successfully intercepted in control and cued conditions for each group. NT neurotypical; ASD autism spectrum disorder. *Error bars* indicate Bias-Corrected Accelerated bootstrapped 95% confidence intervals (based on 10,000 samples). *denotes significant between-group difference (*p* < .05)

For task performance, analysis revealed a significant main effect of group (*F*(1,42) = 8.44, *p* = 0.01, *η*_*p*_^*2*^ = 0.17, BF_10_ = 7.41), with average interception rates significantly lower in autistic (87.75 ± 14.78%) compared to neurotypical participants (97.22 ± 3.91%; *W* = 379.50, *p* = 0.001, BF_10_ = 13.23; Fig. 3). However, there were no significant condition effects (*F*(1,42) = 0.08, *p* = 0.78, *η*_*p*_^*2*^ < 0.01, BF_10_ = 0.23) or group-by-condition interactions (*F*(1,42) = 0.06, *p* = 0.81, *η*_*p*_^*2*^ = 0.001, BF_10_ = 0.28). AQ scores negatively correlated with interception rate in both control (*Rs* = − 0.34, *p* = 0.02, BF_10_ = 2.69) and cued (*Rs* = − 0.41, *p* = 0.01, BF_10_ = 13.67) conditions. Conversely, IUS-S values were not significantly associated with task performance in either block of trials (control: *Rs* = − 0.13, *p* = 0.42, BF_10_ = 0.28; cued: *Rs* = − 0.17, *p* = 0.26, BF_10_ = 0.41).

**Fig. 4:**
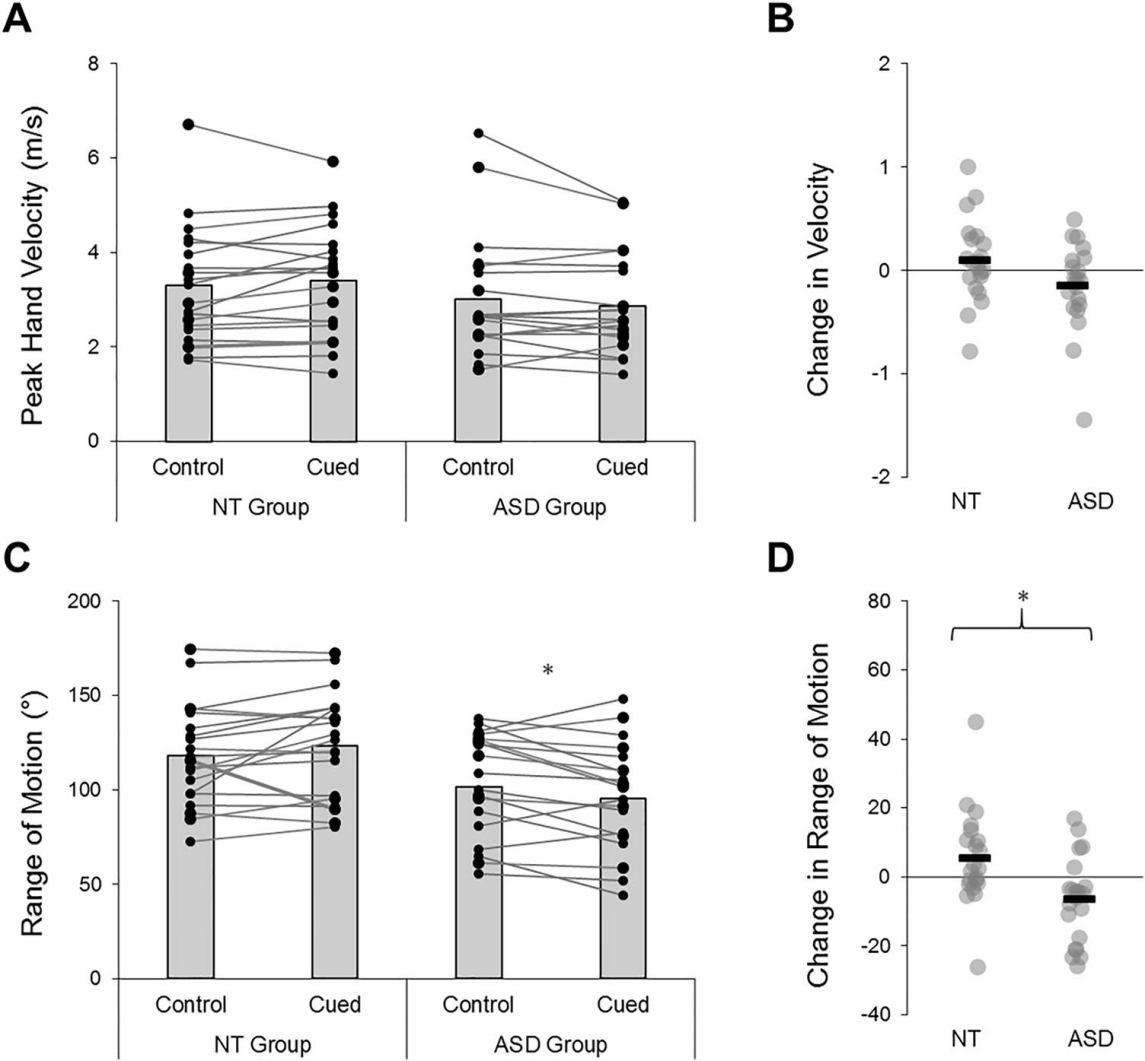
Average peak hand velocities (**A**) and range of motion (**C**) during foreswing actions. Between-condition changes are illustrated in panels (**B**, **D**). NT neurotypical; ASD autism spectrum disorder; *Denotes significant difference (*p* < .05)

There were no significant main effects of group (*F*(1,40) = 1.47, *p* = 0.23, *η*_*p*_^*2*^ = 0.0.04, BF_10_ = 0.70) or condition (*F*(1,40) = 0.13, *p* = 0.73, *η*_*p*_^*2*^ < 0.01, BF_10_ = 0.23) for peak swing velocity, nor were there any significant interactions for this metric (*F*(1,40) = 3.82, *p* = 0.06, *η*_*p*_^*2*^ = 0.09, BF_10_ = 0.97). In terms of ROM, there was a main effect of group (*F*(1,40) = 7.58, *p* = 0.01, *η*_*p*_^*2*^ = 0.16, BF_10_ = 4.43) and a significant group-by-condition interaction (*F*(1,40) = 8.88, *p* = 0.01, *η*_*p*_^*2*^ = 0.18, BF_10_ = 7.50). Autistic participants exhibited lower ROM than neurotypical participants (see Fig. 4). However, while these individuals generally decreased ROM between control and cued conditions (Mean difference: − 6.50 ± 12.38°; *t*(20) = 2.41, *p* = 0.03, BF_10_ = 2.30), neurotypical values remained relatively stable (Mean difference: 5.50 ± 13.68°; *t*(20) = 1.84, *p* = 0.08, BF_10_ = 0.95). ROM significantly correlated with AQ scores in the cued (*R* = − 0.39, *p* = 0.01, BF_10_ = 4.38) but not the control trials (*R* = − 0.25, *p* = 0.11, BF_10_ = 0.66). Moreover, ROM negatively associated with IUS-S scores in both conditions (control: *R* = − 0.42, *p* = 0.01, BF_10_ = 8.40; cued: *R* = − 0.42, *p* = 0.01, BF_10_ = 7.32). Peak swing velocities did not significantly correlate with AQ or IUS-S scores during either condition (*p’s* > 0.06, all BF_10_ < 2).

### Gaze Responses

**Fig. 5:**
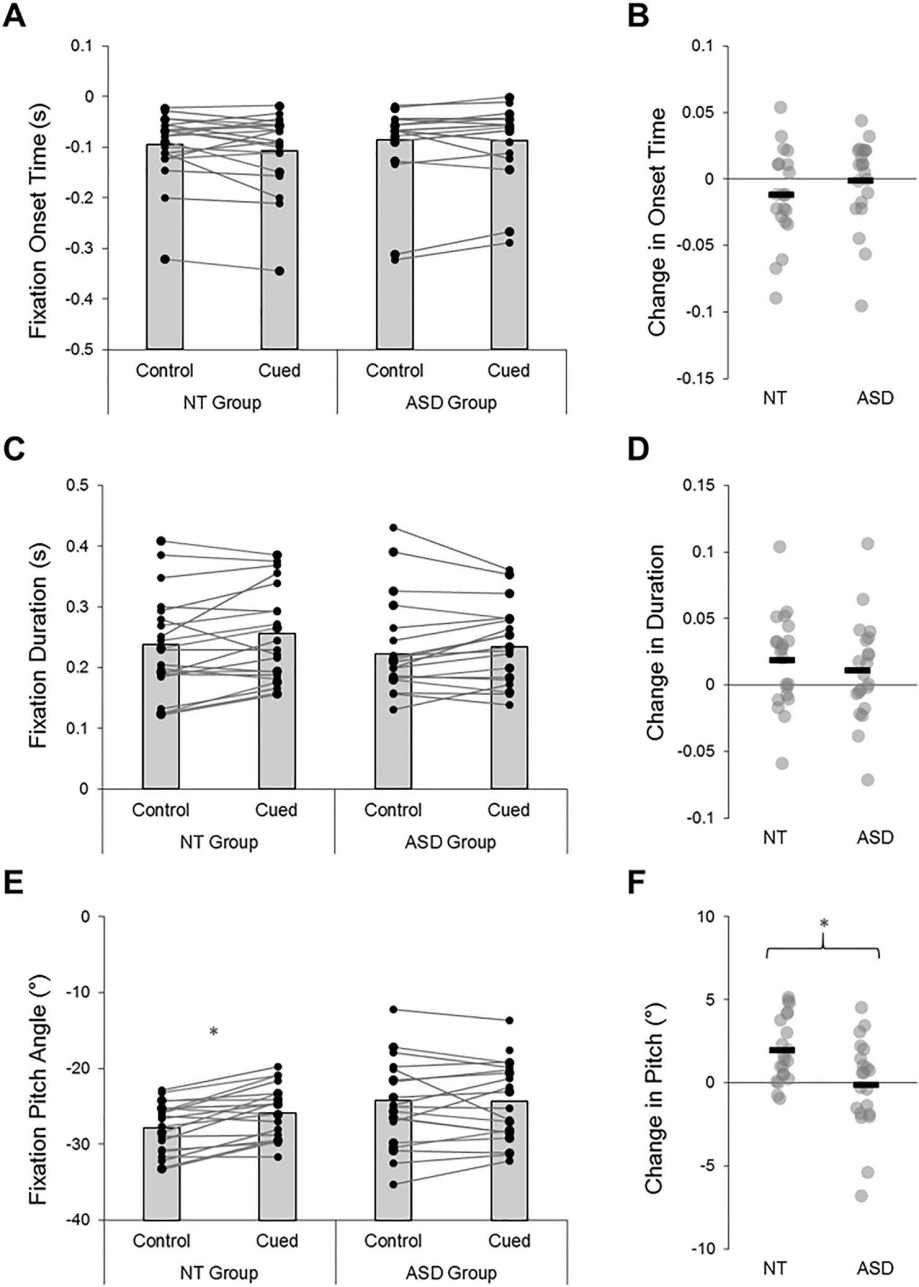
The average onset times (**A**), durations (**C**), and pitch angle (**E**) of predictive bounce fixations. Between-condition changes are illustrated in panels (**B**, **D**, and **F**). NT neurotypical, ASD autism spectrum disorder; *Denotes significant differences (*p* < .05)

Groups exhibited similar gaze profiles during the task. ANOVAs revealed no significant group differences or group-by-condition interactions in relation to the onset and duration of predictive bounce fixations (*p*’s > 0.30; all BF_10_ < 0.67). Participants maintained slightly longer fixations during the cued trials (Fig. 5), with a significant effect of condition emerging for this metric (*F*(1,40) = 6.72, *p* = 0.01, *η*_*p*_^*2*^ = 0.14, BF_10_ = 3.49). However, this main effect did not emerge in relation to onset time (*F*(1,40) = 1.56, *p* = 0.22, *η*_*p*_^*2*^ = 0.04, BF_10_ = 0.44), and there were no significant AQ or IUS-S correlations for either fixation metric (*p*’s > 0.18; all BF_10_ < 0.67).

Next, the average pitch angle (i.e., vertical position) of participant’s predictive bounce fixation was examined. Here, both a significant main effect of condition (*F*(1,40) = 6.39, *p* = 0.02, *η*_*p*_^*2*^ = 0.14, BF_10_ = 2.10) and a significant group-by-condition interaction emerged (*F*(1,40) = 7.92, *p* = 0.01, *η*_*p*_^*2*^ = 0.17, BF_10_ = 5.32). Average pitch values generally increased from control to cued trials, however these changes were group-dependent (see Fig. 5). Specifically, neurotypical participants elevated the height of their bounce fixations after receiving explicit probabilistic cues (Mean difference: 1.96 ± 1.93°; *t*(20) = 4.64, *p* < 0.001, BF_10_ = 184.36), whereas autistic participants showed minimal changes between blocks (Mean difference: 0.11 ± 2.75°; *t*(20) = 0.18, *p* = 0.86, BF_10_ = 0.23). Surprisingly though, no statistical relationships emerged between bounce fixation pitch angles and scores on the AQ or IUS-S (*p*’s > 0.19; all BF_10_ < 0.50).

**Fig. 6:**
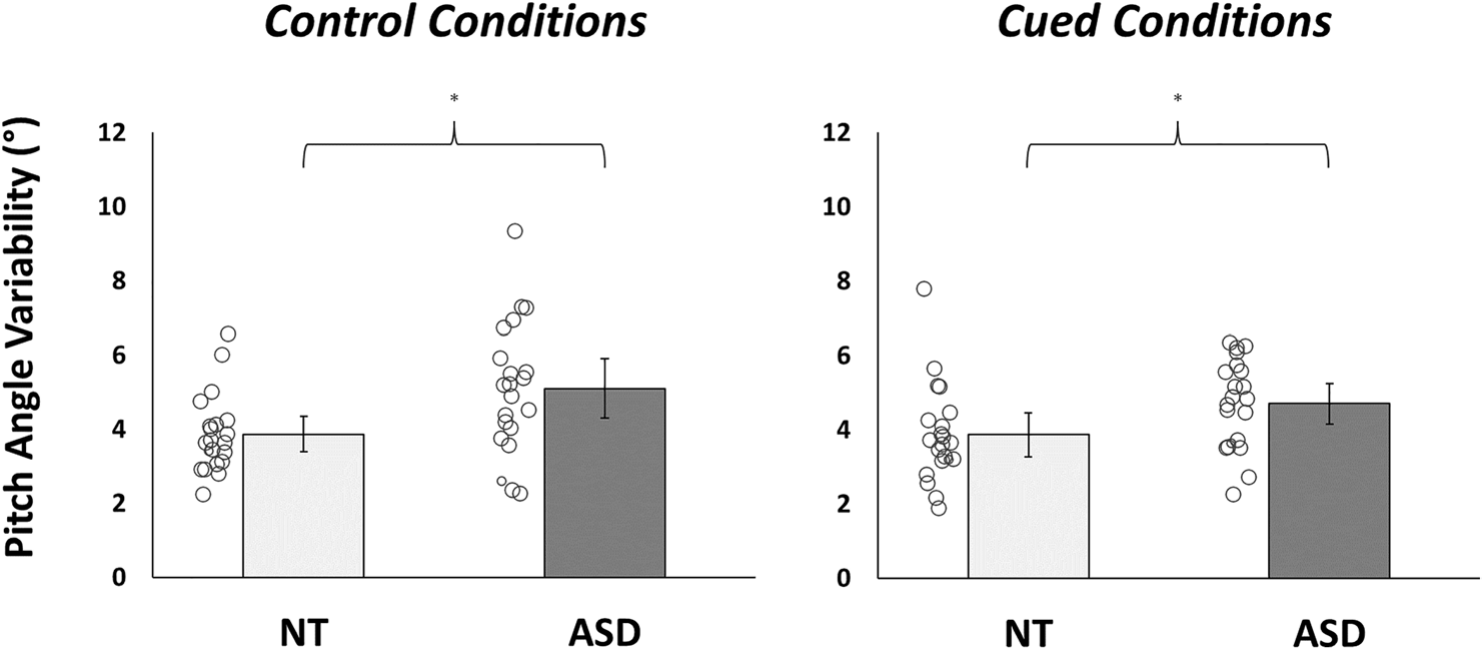
Trial-by-trial standard deviations in the pitch angle of predictive bounce fixations during control and cued blocks. NT neurotypical; ASD autism spectrum disorder; *Denotes significant differences (*p* < .05)

Finally, the trial-to-trial variability in participant’s predictive bounce fixation location was examined. This analysis revealed a significant main effect of group (*F*(1,40) = 6.99, *p* = 0.01, *η*_*p*_^*2*^ = 0.15, BF_10_ = 4.63). Generally, autistic participants showed higher pitch angle SDs than their neurotypical counterparts (Fig. 6). However, these variability scores did not significantly differ between conditions (*F*(1,40) = 1.69, *p* = 0.20, *η*_*p*_^*2*^ = 0.04, BF_10_ = 0.46), and there were no significant group-by-condition interactions (*F*(1,40) = 1.75, *p* = 0.19, *η*_*p*_^*2*^ = 0.04, BF_10_ = 0.57). Furthermore, no statistical associations emerged between pitch angle SD, AQ totals, and IUS-S scores (*p*’s > 0.12, all BF_10_ < 0.67).

## Discussion

This study examined the effects of explicit contextual cues on autistic sensorimotor behaviours during a VR-based interceptive racquetball task. Previous research has shown that prior situational information about likely task outcomes can enhance neurotypical action responses (Gray, 2015; Gredin et al., 2018; Navia et al., 2013), and it has been suggested that such an approach could help autistic individuals in uncertain sensory environments (Haker et al., 2016; Qian & Lipkin, 2011). Therefore, it was hypothesised that autistic people would display improved task performances and enhanced predictive sensorimotor control under explicit cue conditions in this study.

Contrary to these initial hypotheses, we found no significant effects of the explicit contextual cues on interceptive task performances. Lower interception rates and more restricted swing kinematics were evident in autistic sensorimotor responses (Figs. 3, 4), irrespective of any prior probabilistic information. Such impaired motor responses replicate previous findings in this task (Arthur et al., 2021b) and in a host of other empirical assessments (e.g., Chen et al., 2019; Green et al., 2002; Whyatt & Craig, 2013). Crucially though, results indicated that there were no generic difficulties in understanding the VR-based contextual cues in our study (see *Supplementary Analyses*). Indeed, it has already been demonstrated that autistic people can use explicit situational information to guide perceptual and motor abilities (Balconi et al., 2012; Fulceri et al., 2018; Gowen et al., 2020; Soroor et al., 2021; Thillay et al., 2016; Vermeulen, 2015). Instead, null findings suggest that autistic people simply did not benefit from the explicit contextual information that was afforded to them in this task.

The contextual cues in this study provided explicit and veridical information about environmental uncertainty, in a manner that was designed to enhance dynamic state predictions. However, contrary to our hypotheses, we found no evidence for enhanced predictive sensorimotor control under the cued experimental trials. Indeed, while neurotypical participants used the explicit contextual cues to adjust their gaze fixation behaviours (i.e., they directed them to higher spatial locations), autistic individuals showed minimal between-condition changes in their prediction-related visual sampling responses (Fig. 5). Though surprising, these findings align with observations that certain prediction-related atypicalities in autism persist in the face of veridical visual cues about likely trial outcomes (Balsters et al., 2017; Cannon et al., 2021; Greene et al., 2019; Thillay et al., 2016). In fact, the autism group appeared to *restrict* swing ROM under cued conditions (Fig. 4), a response which typically coincides with heightened uncertainty estimates (Arthur et al., 2021). Results therefore imply that autistic people were over-reactive to both implicit and explicit cues about environmental volatility, causing them to employ visuomotor behaviours that are typically affiliated with imprecise (i.e., uncertain) higher-level beliefs.

Taken together, our findings support proposals that autistic sensorimotor control is underpinned by atypicalities in the dynamic modulation of prediction error (e.g., Friston et al., 2013; Lawson et al., 2014; Van de Cruys et al., 2014). Specifically, autistic people were able to detect environmental changes and learn implicit cue-outcome relationships (see also Manning et al., 2017; Sapey-Triomphe et al., 2021); however, their trial-by-trial weighting and updating of action predictions proved to be suboptimal. According to predictive coding perspectives, the context-sensitive mechanisms that regulate synaptic gain signalling (and thus, the weighting of top-down and bottom-up activity) may be atypical in autistic people, leading to a pathologically high receptiveness to sensory inputs and an over-reactivity to contextual change (see Lawson et al., 2017). Such proposals are consistent with our data, which imply that autistic people show a heightened responsivity to recent unexpected events. Indeed, compared to neurotypical individuals, autistic participants directed predictive bounce fixations towards higher spatial locations (Fig. 5) and updated these behaviours more variably from trial to trial (Fig. 6). These profiles reflect an increased tendency to prepare for probabilistically salient and/or volatile events, which directly replicates the findings of our previous work (Arthur et al., 2021b) and in Lawson et al. (2017).

Nonetheless, there was substantial inter-individual variability observed in the dataset. Figures 3–6 illustrate diverging responsivity to contextual cues and levels of task performance, with such heterogeneity proving particularly prominent in autistic individuals. These wide-ranging data patterns are consistent with clinical research (Coll et al., 2020; Fournier et al., 2010), and suggest that sensorimotor difficulties may bear varied aetiologies and neurobiological underpinnings. This notion is not at odds with predictive processing frameworks, as synaptic gain control is theoretically underpinned by a myriad of interacting networks and modulatory systems (see Lawson et al., 2014). Future work must consider these heterogeneous individual aetiologies when attempting to reduce sensorimotor difficulties through applied interventions.

Notably, relationships between sensorimotor control and intolerance of uncertainty were trivial in this study. IUS-S scores did not significantly correlate with any of our performance or visual sampling metrics. These null effects are perhaps unsurprising, as associations between intolerance of uncertainty and anxiety are mechanistically distinct from those concerning hierarchical state estimates and predictions (Bervoets et al., 2021). Indeed, the IUS-S indexes an individual’s chronic disposition to appraise uncertain outcomes as aversive (Carleton et al., 2007), which sits in stark contrast to the highly dynamic and context-sensitive predictive behaviours that were assessed in our virtual racquetball task. Nevertheless, intolerance of uncertainty may affect key moderators of sensorimotor development, such as an individual’s affective state, attention, confidence, and participation in physical activity behaviours (Del Popolo Cristaldi et al., 2021; Robinson & Freeston, 2015). As such, one must not overlook the potential contribution that the construct plays in more applied daily living skills.

From a practical perspective, many autism researchers advocate the provision of explicit contextual information about the underlying statistical properties of a task (e.g., Gomot & Wicker, 2012; Haker et al., 2016; Qian & Lipkin, 2011; Vermeulen, 2015). Though clearly beneficial in many settings, our data suggest that this approach is not necessarily appropriate for developing sensorimotor skills that are inherently changeable and unpredictable in nature. Instead, results support strategies that address the implicit, heterogeneous difficulties that many autistic people face when processing dynamic sensory cues. To alleviate these difficulties, practitioners could look to make task environments feel more predictable for autistic people (e.g., through reducing external sensory ‘noise’, developing individualised routines, or increasing learning repetition blocks; see Haker et al., 2016). Similarly though, practitioners should also look to help individuals deal with volatile and unpredictable elements of sensorimotor skills. Indeed, personalised task modifications may not always be possible at a practical level, and so future work could focus on developing strategies that facilitate the sampling of ‘optimal’ sensory cues (e.g., see feedforward gaze training: Wilson & Vine, 2018; environmental scaffolding: Van de Cruys et al., 2014).

Nevertheless, there are some key limitations that should be considered in future work. Firstly, our results are based on indirect measures of sensorimotor prediction, which were taken from a relatively small number of trials. Studies suggest that changes in prior contextual beliefs can be detected within ten repetitions (Verstynen & Sabes, 2011), however future investigations may wish to examine longer-term adaptations in predictive sensorimotor control and learning (as in Lawson et al., 2017; Vossel et al., 2014). Moreover, future work could incorporate self-rating methods that directly index participants’ trial-by-trial predictions (e.g., Pasturel et al., 2020). Secondly, despite being unconstrained and naturalistic in design, the racquetball task was performed under tightly-controlled VR conditions. Hence, some potentially significant factors that contribute to ‘real-world’ sensorimotor issues may have been overlooked (e.g., access to support, social/developmental differences). We recommend that future research explores how applied daily living skills can be enhanced in autistic people, especially in activities that are deemed important or challenging for neurodivergent populations (e.g., driving or occupational skills; see Robledo et al., 2012). Finally, the present study did not conduct any standardised assessments for intellectual disabilities or clinically-related cognitive impairments. It must be stressed here, that the link between these factors and autistic movement abilities is yet to be reliably established (Coll et al., 2020; Fournier et al., 2010) and that our participants reported no co-occurring disabilities that are known to affect sensorimotor control. Furthermore, participants did not display any discernible impairments relating to the understanding of explicit contextual cues in VR, as shown in our *Supplementary Information*. As such, it seems unlikely that intellectual abilities will have had any meaningfully confounding effects on our observed results.

In sum, the present research found that autism-related sensorimotor difficulties are not alleviated through the provision of explicit contextual cues about environmental uncertainty and stability. Although studied in the unique context of an interceptive VR racquetball task, results indicate that sensorimotor issues are unlikely to reflect any generic impairments in the ability to detect changing environmental probabilities (or any broad intolerance of uncertainty). Instead, results imply that the context-sensitive modulation of top-down predictions and bottom-up sensory cues is atypical in autism. It is therefore recommended that practitioners look to help autistic people build stable action predictions that can help guide movement skills and learning, possibly through the use of inclusive environmental adjustments and/or individualised learning methods.

### Supplementary Information

Below is the link to the electronic supplementary material.

Supplementary file1 (DOCX 21 kb)

## Data Availability

Materials, customised code, and data from the current study are publicly available at: https://osf.io/5y48g/.
