# Supplementary material for: Investigating how Explicit Contextual Cues Affect Predictive Sensorimotor Control in Autistic Adults
Source: J Autism Dev Disord. 2022 Sep 5;53(11):4368–81. doi: 10.1007/s10803-022-05718-5 (PMC10539449; doi:10.1007/s10803-022-05718-5)
Supplement: Supplementary file 1 — Supplementary file1 (DOCX 21 kb) [file 10803_2022_5718_MOESM1_ESM.docx]

**Supplementary Information**

***Exploratory Analysis of Gaze Fixation Data***

Follow-up tests explored whether group-dependent changes in gaze behaviours derive from altered volatility processing or whether they are simply reflecting participants’ ability to use prior probabilistic information. For instance, the cued block contained periods of 6-12 trials where the likelihood of facing a bouncy ball was described as ‘high’ to participants. Thus, it is possible that higher pitch averages in cued versus control trials are being driven by data from these specific datapoints. Conversely, autistic participants may have difficulties interpreting these cues, leading to non-significant changes between conditions. In the analysis that follows, data from selected trials are scrutinised within each condition, with two participants and their matched counterparts excluded due to missing outcome values (remaining *n* = 40).

Firstly, gaze data from trials that immediately followed a ‘high’ probabilistic cue were extracted for each participant. Prior to these trials, participants viewed a ‘hawkeye’ illustration which projected an equal probability of facing a normal or bouncy ball (see Figure 1). This was accompanied by an indication that the current likelihood of facing a bouncy ball was relatively ‘high’. If these probabilistic cues were being readily used by participants, then one would expect subsequent predictive bounce fixations to be higher than control values. Indeed, a mixed-model ANOVA found a significant effect of condition on the extracted fixation data (*F*(1,38) = 50.15, *p* < .001, *η_p_^2^* = 0.57; BF_10_ = 2.67×10^5^), with both groups exhibiting increases in the height of their predictive gaze fixations (Wilcoxon Signed-Rank: *Z* = 5.51, *p* < .001, BF_10_ = 4.76×10^5^). Importantly, no significant interactions were recorded (*F*(1,38) = 2.99, *p*= .09, *η_p_^2^* = 0.07; BF_10_ = 0.91).

Next, mixed-model ANOVAs were repeated using data from catch trials *only*. In these trials, no prior information about likely ball bounciness was provided; participants were simply cued that the environment had changed. Notably, the significant interaction effects observed in our main analyses were replicated in this data (*F*(1,38) = 5.24, *p* = .03, *η_p_^2^* = 0.12), albeit with weaker statistical strength (BF_10_ = 2.40). Together, results suggest that group-dependent changes in visual sampling behaviour were not due to differences in the interpretation or understanding of our explicit VR cues. Instead, they appear related to expectations about environmental stability (see *main discussion*).
